# Protocol for a prospective, randomized study on neurophysiological assessment of lower urinary tract function in a healthy cohort

**DOI:** 10.1186/s12894-016-0188-9

**Published:** 2016-11-25

**Authors:** Stéphanie van der Lely, Martina Stefanovic, Melanie R. Schmidhalter, Marta Pittavino, Reinhard Furrer, Martina D. Liechti, Martin Schubert, Thomas M. Kessler, Ulrich Mehnert

**Affiliations:** 1 Neuro-Urology, Spinal Cord Injury Center & Research, University of Zürich, Balgrist University Hospital, Forchstrasse 340, 8008 Zürich, Switzerland; 2 Institute of Mathematics, University of Zürich, Winterthurerstrasse 190, 8057 Zürich, Switzerland; 3 Neurophysiology, Spinal Cord Injury Center & Research, University of Zürich, Balgrist University Hospital, Forchstrasse 340, 8008 Zürich, Switzerland

**Keywords:** Sensory evoked potential, Electroencephalography, Lower urinary tract, Urinary bladder, Urethra, Randomized, Lower urinary tract dysfunction, Current perception threshold, A-delta afferent fibers, Electrical stimulation

## Abstract

**Background:**

Lower urinary tract symptoms are highly prevalent and a large proportion of these symptoms are known to be associated with a dysfunction of the afferent pathways. Diagnostic tools for an objective and reproducible assessment of afferent nerve function of the lower urinary tract are missing. Previous studies showed first feasibility results of sensory evoked potential recordings following electrical stimulation of the lower urinary tract in healthy subjects and patients. Nevertheless, a refinement of the methodology is necessary.

**Methods:**

This study is a prospective, randomized trial conducted at Balgrist University Hospital, Zürich, Switzerland. Ninety healthy subjects (forty females and fifty males) without lower urinary tract symptoms are planned to be included in the study. All subjects will undergo a screening visit (including standardized questionnaires, 3-day bladder diary, urinalysis, medical history taking, vital signs, physical examination, neuro-urological examination) followed by two measurement visits separated by an interval of 3 to 4 weeks. Electrical stimulations (0.5Hz-5Hz, bipolar, square wave, pulse width 1 ms) will be applied using a custom-made transurethral catheter at different locations of the lower urinary tract including bladder dome, trigone, proximal urethra, membranous urethra and distal urethra. Every subject will be randomly stimulated at one specific site of the lower urinary tract. Sensory evoked potentials (SEP) will be recorded using a 64-channel EEG cap. For an SEP segmental work-up we will place additional electrodes on the scalp (Cpz) and above the spine (C2 and L1). Visit two and three will be conducted identically for reliability assessment.

**Discussion:**

The measurement of lower urinary tract SEPs elicited by electrical stimulation at different locations of the lower urinary tract has the potential to serve as a neurophysiological biomarker for lower urinary tract afferent nerve function in patients with lower urinary tract symptoms or disorders. For implementation of such a diagnostic tool into clinical practice, an optimized setup with efficient and reliable measurements and data acquisition is crucial. In addition, normative data from a larger cohort of healthy subjects would provide information on variability, potential confounding factors and cut-off values for investigations in patients with lower urinary tract dysfunction/symptoms.

**Trial registration:**

Clinicaltrials.gov; Identifier: NCT02272309.

## Background

Lower urinary tract symptoms (LUTS) such as urinary urgency, frequency and incontinence, imply a massive impairment of quality of life [1, 2].

LUTS are highly prevalent and a large proportion of LUTS are found to be associated with afferent nerve dysfunction [1, 3–5]. Assessment of afferent pathways in patients with LUTS is however a challenge. Specific diagnostic tools for an objective and reproducible measurement of bladder and urethral afferent nerve function are missing. Yet, filling cystometry (FC) is the standard method used in clinical practice for the assessment of bladder sensations [6–10]. Nevertheless, FC largely depends on the subjective perceptions and collaboration of the patient and is hence not an objective measurement of bladder sensations. In addition, the reliability of the FC is questionable and its variability and outcome resolution is too large to detect differences smaller than 100 mL [11, 12]. Moreover, FC only covers sensory information from the bladder but not from the urethra.

Current perception threshold (CPT) testing is another method of assessing sensations from the lower urinary tract (LUT). CPT testing is performed by asking the subject to indicate the onset of sensation when an increasing electrical stimulus is applied [13]. It was shown that this method is safe and well tolerated by healthy subjects and patients, but still, it provides only semi-quantitative information on sensations of the LUT [14–16]. In addition, local factors such as distance of electrodes to the mucosa and the mucosal condition itself can significantly affect CPTs [14, 17, 18].

**Fig. 1:**
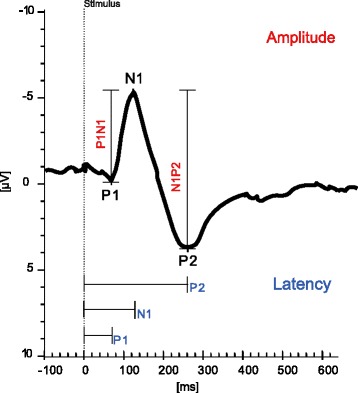
Example of a cortical LUT SEP recorded at Cz with the markers of the P1, N1 and P2 peaks and the corresponding latencies and peak-to-peak amplitudes

A more objective and qualitative assessment of afferent nerve function are sensory evoked potentials (SEP) that are routinely used in neurophysiology to detect afferent nerve conduction qualities and integrity from different parts of the human body. By analysing the latencies and amplitudes of the SEPs (Fig. 1), information on nerve fiber integrity, conduction velocity and fiber type can be obtained [19, 20]. SEPs from the LUT would be useful not only for an assessment of LUT sensory function, to amend findings from previous investigations (i.e. history, neurologic examination, urodynamic examination), but also as a surrogate marker and outcome measure for treatments targeting afferent LUT pathways [21]. However, SEP measurements stimulating the LUT are more challenging than SEP measurements for cutaneous sites due to less direct control of electrode placement and potential changes of bladder volume with time, which can influence the SEP measurement. Furthermore, bladder SEPs may be less synchronized as they are likely mediated by poorly or non-myelinated fibres leading to less distinct summation of sensory potentials. Nevertheless, previous studies reported first feasibility results of SEP recordings from the LUT following electrical stimulation in healthy subjects [22–26] and patients [20, 27–29]. However, due to heterogeneous measurement settings and study populations, a clear conclusion cannot be drawn from these data. Currently, there is no standard for SEP measurements for the LUT. Hence, optimal stimulation and recording procedures as well as parameters still need to be determined.

In this study we would like to advance the evaluation of viscero-sensory afferent pathways of the LUT and to refine the methodology of LUT SEPs in healthy subjects. We aim to get more knowledge on the impact of different stimulation parameters (e.g. stimulation frequency) on the reliability, shape, latency, amplitude and topographical distribution of SEPs recorded during electrical stimulation of the LUT. In a first step it is our goal to find a frequency that allows a faster acquisition of reliable SEPs than the previously used 0.5 Hz [25, 26] and to obtain normative LUT SEP data for the different localizations in the LUT from different gender groups. In a second step we aim to implement the optimized methodology into clinical practice to use it as an objective marker of pathological LUT conditions and to show distinction of healthy LUT neurophysiology and function.

## Methods and design

### Study design

**Fig. 2:**
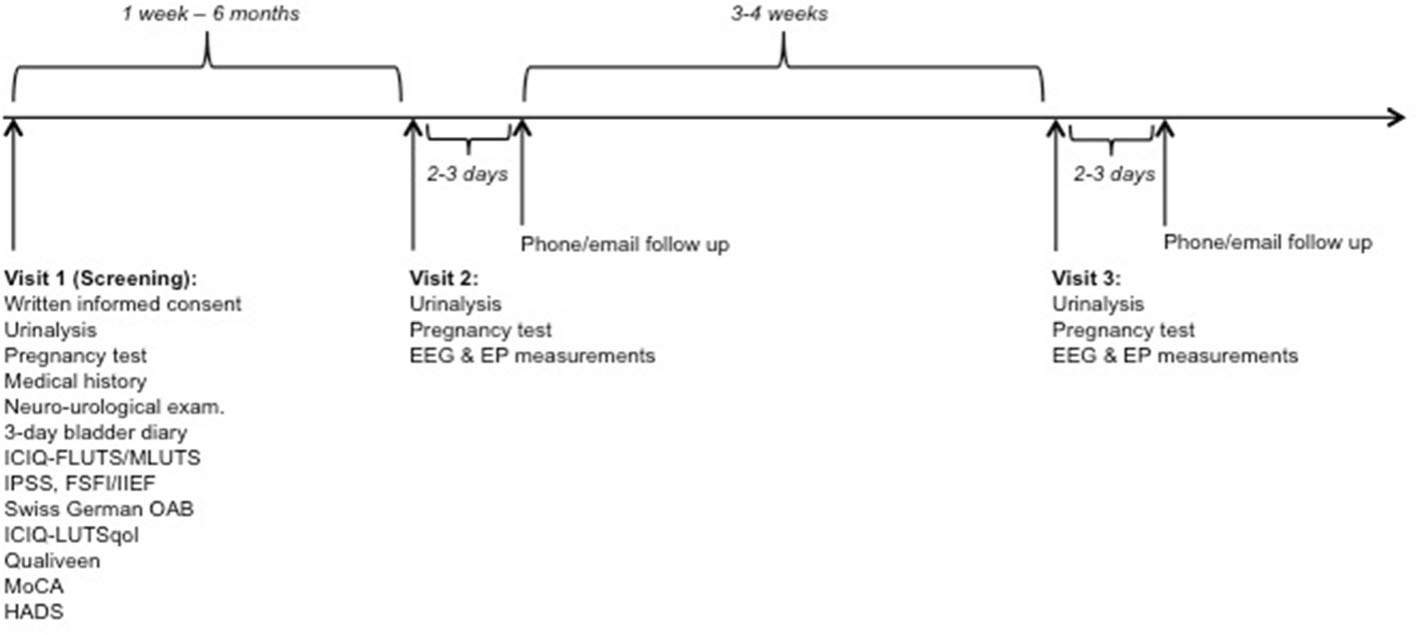
Study design and time schedule

This study is a prospective, randomized trial conducted as a single center study at the Spinal Cord Injury Center & Research Lab, Balgrist University Hospital, University of Zürich, Zürich, Switzerland. The study comprises three visits of which the first will be a screening visit followed by two measurement visits separated by an interval of 3–4 weeks (Fig. 2). The site of stimulation will be indicated by random group assignment (in females and males: dome, trigone, proximal urethra, distal urethra; in males additionally: membranous urethra). Consequently, ten females and ten males will be allocated to one localization. In addition, the frequencies used for the stimulation of the LUT and thereafter the SSEP measurements will be randomly applied.

### Study population and recruitment

The volunteers will be recruited via announcements at the University of Zürich, internet platforms (i.e. www.marktplatz.uzh.ch, www.tutti.ch, www.ronorp.net) and personal contacts. According to the inclusion- and exclusion criteria (Table 1), healthy female and male subjects without any LUTS will be included. Health is defined as the absence of any health troubles, as assessed by a complete medical history, standardized questionnaires, physical, neurological and neuro-urological examinations (Table 2). The absence of LUTS will be determined by uroflowmetry, a 3-day bladder diary and standardized urological questionnaires (FLUTS [30] /MLUTS [31]; Qualiveen [32]; IPSS [33]; Swiss German OAB [34]) (Table 2).

**Table 1:** Inclusion and exclusion criteria for healthy female and male volunteers

Inclusion criteria	Exclusion criteria
Written informed consent	Any lower urinary tract symptoms^d, f, c^
Good mental^a^ and physical health^c^	Any neurological or urological pathology ^e, f, c^
Age > 18 years^c^	Current pregnancy^bc^, lactation^c^
No regular intake of medication^c^	UTI^b, c, f^
Bladder capacity <150 mL or SDV already at 60mL^d, e, g^	Hematuria^b^
Number of voids per day <8, number of voids per night <2^d, f, c^	Any anatomical anomaly/malignancy of the LUT or genitalia ^e, c^
	Any previous pelvic, spine or craniocerebral surgery^c^

*LUT* lower urinary tract, *SDV* strong desire to void, *UTI* urinary tract infection

^a^assessed by MoCA and HADS; ^b^excluded by urine dipstick test; ^c^assessed by history taking; ^d^assessed by 3-day bladder diary; ^e^assessed by neuro-urological examination ^f^assessed by standardized urological questionnaires (FLUTS, MLUTS, IPSS, Qualiveen, Swiss-German OAB questionnaire); ^g﻿^assessed by uroflowmetry

**Table 2:** Overview of the screening procedure

Tests	Questionnaires / Bladder diary
Urine dipstick	FLUTS [28] / MLUTS [29]
Pregnancy	Qualiveen [30]
	ICIQ-LUTSqol [35]
	IPSS [31] (only for males)
**Examinations**	Swiss German OAB questionnaire (short form) [32]
Bulbocavernosus reflex	FSFI [34] / IIEF [33]
Anal reflex	HADS [36]
Pin-prick of S2-S5 dermatomes	MoCA [37]
Uroflowmetry	3-day bladder diary
Assessment of medical history	
Physical examination	

### Investigations and procedures

#### Screening (visit one)

The content and purpose of the study will be explained in written and oral form to all recruited subjects. Those subjects providing written informed consent will be screened for in- and exclusion criteria by using the tests, questionnaires, and examinations listed in Table 2. Subjects who are eligible for study participation according to the in- and exclusion criteria (Table 1) will be invited for visit two and three (Fig. 2).

#### Measurement visits (visit two and three)

**Fig. 3:**
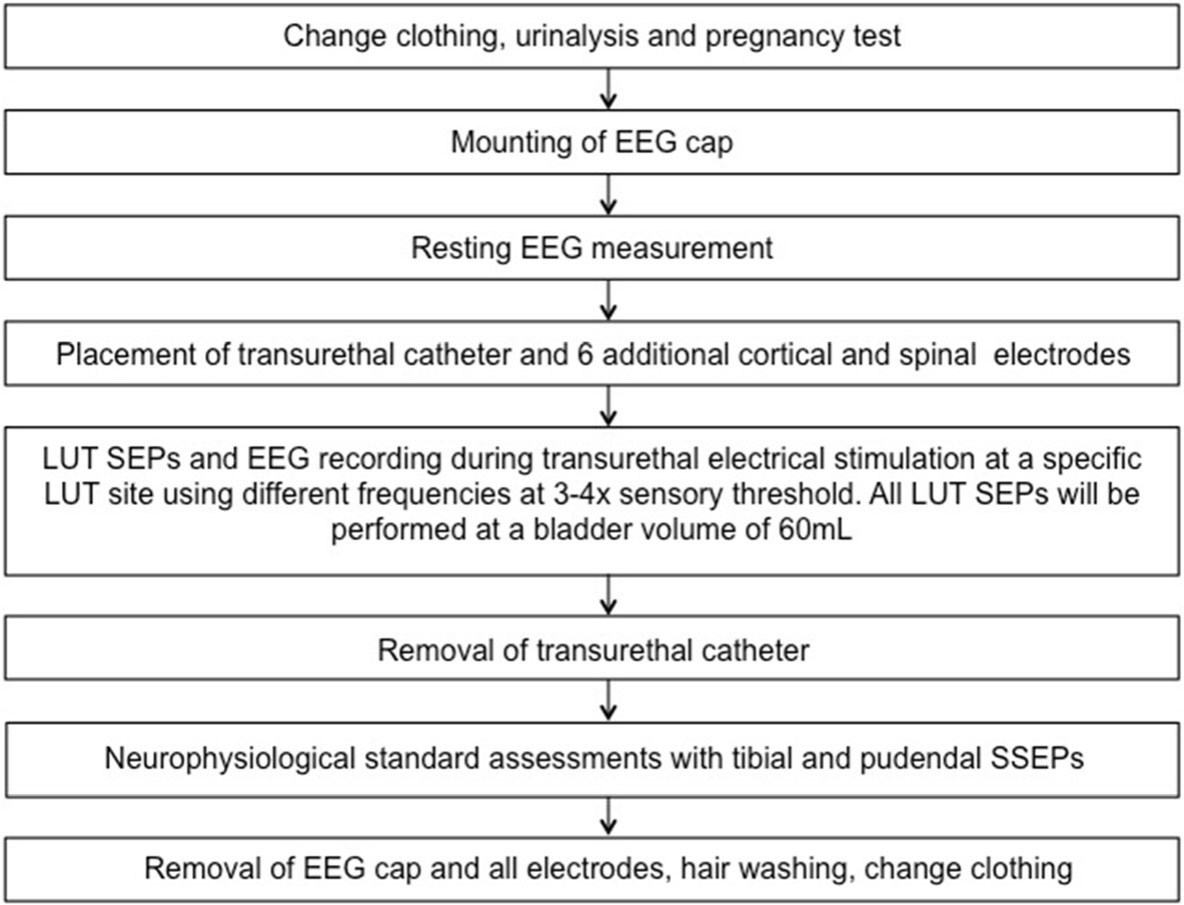
Flowchart of the measurement visits (visits two and three)

Prior to each measurement, the urine of the volunteers is analyzed to exclude signs suggestive for asymptomatic bacteriuria, microhaematuria, and (in women only) pregnancy. Both measurements consist of a resting electroencephalogram (EEG) measurement followed by recordings of SEPs elicited by transurethral electrical stimulation at a specific LUT site indicated by the group assignment. Each measurement includes recordings of the electrooculogram (left and right eye), electrocardiogram, and electroencephalogram using a 64 Ag/AgCl surface electrodes system comprising a cap-based extended international 10–20 montage (Easy cap, Easy cap GmbH, Herrsching, Germany). Electrode impedances are constantly kept below 20kΩ. Six additional electrodes are placed at Cpz (reference: Fz), C2 (reference: Fz) and L1 (reference: iliac crest), respectively, for segmental assessment. LUT electrical stimulation will be applied transurethrally with a custom-made 14 Ch catheter (Unisensor AG, Attikon, Switzerland), using frequencies between 0.5Hz and 5Hz (bipolar, square wave, pulse width 1 ms). Stimulation intensities are adapted to the 3 to 4× CPT, which is determined using the method of limits prior to each SEP measurement [13]. The catheter includes platinum electrodes and a radiopaque marker, which allows precise catheter positioning under fluoroscopic guidance. After each stimulation, the bladder will be emptied and filled with 60 mL of contrast medium (Ultravist® 150^TM^, Bayer AG, Switzerland). Consequent to LUT SEPs, SEPs elicited by transcutaneous stimulation of the tibial and pudendal nerves will be recorded in random order. These standard neurophysiological measurements will serve as comparators to the LUT SEPs. Visits two and three will be performed identically with an interval of 3 to 4 weeks (Fig. 3).

#### Follow-up

Two to three days after each measurement visit, a follow-up interview is performed to evaluate the general well-being of the volunteers. In case of any side effects, such as dysuria, the subjects are appointed to an extra medical visit for further evaluation, investigation and, if necessary, medical treatment.

### Safety

During the first visit, all subjects are carefully screened to exclude subjects with neurological and/or urological pathology or any regular medication intake. At the beginning of every visit an urinalysis and pregnancy test (in females only) is performed to exclude signs suggestive for asymptomatic bacteriuria, microhaematuria and pregnancy, respectively. Pregnancy leads to study exclusion and referral to a gynecologist for further evaluation. In case of a positive urine dipstick test suspicious for asymptomatic bacteriuria or urinary tract infection (UTI), the measurement will be postponed until the dipstick test result becomes negative or UTI has been treated. In case of a dipstick test indicating microhaematuria, subjects can choose to repeat the test at a later time-point or to directly have the result verified by clean catheterization. If clean catheterization still indicates microhaematuria, subjects will be excluded from study participation and referred to their general practitioner or urologist for further evaluation.

Two or three days after the measurement visits, a follow-up interview will be conducted to assess general well-being and document possible adverse events or symptoms. In case of an adverse event, additional tests or medical interventions will be initiated as necessary and subjects might be referred to a general physician or medical specialist for further investigations and/or treatment. All responsible authorities will be informed about any adverse events (AE) or severe adverse events (SAE). These events will be observed and followed until complete cure. To decrease the radiation dose during visits two and three, we will not perform full radiographs but fluoroscopy with a reduced field of view focusing on the LUT only. For a protection of the male gonads, men will wear a gonad shielding.

### Endpoints of the study

Primary endpoint: N1 responder rate / latency of N1 – as the most prominent peak of LUT SEPs.

Secondary endpoints: A) Latencies (P1, P2), amplitudes (P1, N1, P2, P1N1, N1P2), topographies and source localizations of LUT SEPs; B) CPTs; C) latencies, amplitudes, topographies and source localization of tibial, pudendal SSEPs; D) 3-day bladder diary, scores of questionnaires (i.e. ICIQ-FLUTS [30]/ICIQ-MLUTS [31], IPSS [33], IIEF [35] /FSFI [36], Swiss German OAB [34], ICIQ-LUTSqol [37], Qualiveen [32], HADS [38], MoCA [39]).

### Determination of sample size

Based on data from a previous study [25], the sample size was determined using a non-parametric approach [40]. Non-parametric smoothing estimates (kernel smooth) were iteratively compared for subsets of individuals in order to establish the smallest subset (sample size) in which a significant outcome was observed. Determined using cross-validation [41], the smoothing parameter was set to 20. From the smoothed curves, the empirical second derivatives (as an expression of the 'information', i.e. latency, amplitude and dispersion, contained therein) were used, the estimates of which were computed and standardized by the absolute value of their mean. The difference between the two frequencies (0.5Hz and 3Hz) was taken into account, to allow for a standardized vector summary of the curve. Normality was tested using the Kolmogorov-Smirnov method and no evidence against was revealed. A *t*-test based on this standardized vector was subsequently conducted. Power analysis was performed using a bootstrapping technique [42, 43]. For each different sample size combination, the difference between the two frequencies, contained in the standardized vector, was analyzed. The bootstrap simulations were executed at the individual level. All elements of the standardized vector summary were first sub-sampled before results were presented based on the mean p-values for all possible combinations. To ensure robustness of the results, four different criteria – standard deviation, variance, total variance (sum of the absolute values) and *wigglyness* (sum of the absolute values of the second derivatives) – were applied to the standardized vector. The aforementioned procedure was repeated for the different study visits and simulation sites. This resulted in a required sample size of 50 male and 40 female subjects, taking into account potential dropouts.

### Data management and analysis

All EEG data will be filtered and segmented using Brain Vision Analyzer 2 (Version 2.1.0.327, Brain Products, Gilching, Germany). The segments will be averaged and the P1, N1, P2 latencies as well as the P1N1 and N1P2 amplitudes will be determined.

Study data will be collected and managed using the Research Electronic Data Capture Tool (REDCap, Version 6.12.1, Vanderbilt University) electronic data capture tools hosted at Balgrist University Hospital [44]. REDCap is a secure, web-based application designed to support data capture for research studies, providing 1) an intuitive interface for validated data entry; 2) audit trails for tracking data manipulation and export procedures; 3) automated export procedures for seamless data downloads to common statistical packages; and 4) procedures for importing data from external sources.

Primarily, all data will be examined using exploratory data analysis (EDA) methods and described providing mean and standard deviation (or median and range where appropriate). ANOVAs or independent sample t-tests (or Kruskal-Wallis test or Mann-Whitney tests where appropriate) will be performed to compare participant characteristics between groups or to detect gender differences. Linear mixed effects models will be used to compare the two measurement visits. The level of significance will be 5% (alpha = 0.05). Regression techniques will be taken into account, if needed. All the statistical analyses will be performed with the software RStudio (Version 0.98.1083) [45].

## Discussion

This clinical trial will investigate the effect of several stimulation frequencies at different locations of the LUT. Since it was already shown that SEPs could be reproducibly recorded from the LUT [25], we now aim to optimize the settings to achieve a faster acquisition of reliable SEPs, which is important for implementation into clinical diagnostics and to minimize measurement bias through changes that occur over time such as bladder volume.

The assessment of normative values of LUT SEPs in healthy male and female subjects will give us more knowledge on the variability of LUT SEPs, as well as potential factors that may influence the shape and the reliability of the SEPs. Cut-offs for amplitude and latency values can be defined for future investigations in patients with LUT dysfunction. The advancement of neurophysiological assessment methods for the LUT will significantly influence the evaluation of afferent nerve function in the LUT and has the potential to serve as a clinical diagnostic tool complementary to standard urodynamic investigations. After having refined our methodology, we would like to apply LUT SEPs with the optimized stimulation frequency in different patient groups suffering from LUTS, including patients with spinal cord injury and multiple sclerosis. Established cut-off amplitudes and latency values from this study should then be used to relate LUT symptoms and dysfunction in patients with LUT SEP data, thus amending FC findings with an objective evaluation of afferent LUT nerve function in these disorders.

### Trial status

At the time of manuscript submission, first subjects have been recruited, included and investigated.

## Abbreviations

AE: Adverse event
CPT: Current perception threshold
EDA: Exploratory data analysis
EEG: Electroencephalography / electroencephalogram
FC: Filling cystometry
GCP: Good clinical practice
LUTS: Lower urinary tract symptoms
OAB: Overactive bladder
SAE: Severe adverse event
SDV: Strong desire to void
SEP: Sensory evoked potential
UTI: Urinary tract infection
